# Fractional Flow Reserve Is Not Associated with Inflammatory Markers in Patients with Stable Coronary Artery Disease

**DOI:** 10.1371/journal.pone.0046356

**Published:** 2012-10-16

**Authors:** Jan-Willem E. M. Sels, Ellen H. A. M. Elsenberg, Imo E. Hoefer, Anton Jan van Zonneveld, Johan Kuiper, J. Wouter Jukema, Nico H. J. Pijls, Gerard Pasterkamp

**Affiliations:** 1 Department of Cardiology, Catharina Hospital Eindhoven, Eindhoven, The Netherlands; 2 Laboratory of Experimental Cardiology, University Medical Center Utrecht, Utrecht, The Netherlands; 3 Department of Biomedical Engineering, Eindhoven University of Technology, Eindhoven, The Netherlands; 4 Department of Nephrology, Leiden University Medical Center, Leiden, The Netherlands; 5 Division of Biopharmaceuticals, Leiden University, Leiden, The Netherlands; 6 Department of Cardiology, Leiden University Medical Center, Leiden, The Netherlands; University of Freiburg, Germany

## Abstract

**Background:**

Atherosclerosis is an inflammatory condition and increased blood levels of inflammatory biomarkers have been observed in acute coronary syndromes. In addition, high expression of inflammatory markers is associated with worse prognosis of coronary artery disease. The presence and extent of inducible ischemia in patients with stable angina has previously been shown to have strong prognostic value. We hypothesized that evidence of inducible myocardial ischemia by local lesions, as measured by fractional flow reserve (FFR), is associated with increased levels of blood based inflammatory biomarkers.

**Methods:**

Whole blood samples of 89 patients with stable angina pectoris and 16 healthy controls were analyzed. The patients with stable angina pectoris underwent coronary angiography and FFR of all coronary lesions.

We analyzed plasma levels of cytokines IL-6, IL-8 and TNF-α and membrane expression of Toll-like receptor 2 and 4, CD11b, CD62L and CD14 on monocytes and granulocytes as markers of inflammation.

Furthermore, we quantified the severity of hemodynamically significant coronary artery disease by calculating Functional Syntax Score (FSS), an extension of the Syntax Score.

**Results:**

For the majority of biomarkers, we observed lower levels in the healthy control group compared with patients with stable angina who underwent coronary catheterization.

We found no difference for any of the selected biomarkers between patients with a positive FFR (≤0.75) and negative FFR (>0.80). We observed no relationship between the investigated biomarkers and FSS.

**Conclusion:**

The presence of local atherosclerotic lesions that result in inducible myocardial ischemia as measured by FFR in patients with stable coronary artery disease is not associated with increased plasma levels of IL-6, IL-8 and TNF-α or increased expression of TLR2 and TLR4, CD11b, CD62L and CD14 on circulating leukocytes.

## Introduction

Atherosclerosis is the leading cause of mortality and morbidity in the Western world and coronary artery disease is its most prevalent manifestation. Atherosclerosis has been recognized as a chronic inflammatory disease. The influx of inflammatory cells into the vascular wall and release of pro-inflammatory substances drives plaque initiation and progression. In addition, local inflammation is the key factor in the biological events leading to plaque rupture and subsequent thrombosis, causing myocardial infarction and stroke. Ruptured plaques have been shown to be infiltrated with inflammatory cells [1]. This observation initiated the search for systemically expressed markers of inflammation that could reflect the severity and stability of the atherosclerotic disease. As a result increased levels of pro-inflammatory biomarkers have been measured in plasma and on blood cells of patients with acute coronary syndromes [2]–[4]. Furthermore, higher levels of these cytokines have been associated with impaired prognosis in patients with acute coronary syndromes [5], [6].

The detrimental effect of increased inflammatory activity also applies to stable atherosclerotic disease and even asymptomatic individuals. In a healthy cohort, increased levels of interleukin (IL) -6 were associated with increased occurrence of myocardial infarction and coronary death [7]. In another study, IL-8 was predictive for the occurrence of cardiovascular events in patients with stable coronary artery disease [8]. The presence and extent of inducible ischemia is a strong prognostic factor in stable coronary artery disease. It has consistently been shown, both invasively and non-invasively, that coronary artery lesions that give rise to ischemia negatively affect prognosis. On the other hand, if coronary stenoses do not cause ischemia, prognosis is excellent with low event rates [9]–[12]. It is unknown if the inflammatory activity in patients at risk is explained by the local functional severity of a lesion or that it reflects the inflammatory status of the entire vasculature. Considering the reports that both increased inflammatory activity and the presence of inducible ischemia affect the clinical course of stable coronary disease, we hypothesized that functional parameters of locally inducible ischemia and systemic inflammatory parameters are related.

Fractional Flow Reserve (FFR) is an invasive, lesion-specific index of myocardial ischemia and is considered the gold standard for the assessment of ischemic potential of coronary lesions. FFR has a well defined cut-off value for ischemic lesions and has shown excellent reproducibility. FFR-guided treatment of coronary lesions has been shown to be superior to conventional angiography-guided treatment and FFR is of prognostic significance with respect to future acute myocardial infarction or death [11], [12].

In the current study we assessed the relationship of functional coronary lesion significance, assessed by FFR, with both cell-based as well as secreted markers of inflammatory activity.

## Methods

### Patient selection

Our analysis included 89 patients with chronic stable angina that were presented at the catherization lab of the Catharina Hospital Eindhoven, the Netherlands. All patients were investigated for ischemia with FFR.

As a negative control group, 16 healthy individuals aged >40 years without known coronary artery disease or atherosclerosis were included. All participants provided written informed consent prior to participation. This study was approved by the local ethics committee. Exclusion criteria were active inflammatory conditions, autoimmune disease, malignancies, use of immunosuppressive drugs and known hematological disorders. Patients with ST-elevation myocardial infarction were also excluded. Blood of patients with suspected unstable angina or NSTEMI was studied but retrospectively excluded from this analysis.

### Invasive assessment of ischemia by FFR

The technical aspects of FFR measurement have been extensively described previously [13]. In short, a stenosis to be investigated is crossed with a standard pressure-wire (PressureWire^tm^ Certus, St Jude Medical Inc,USA) through a coronary guiding catheter after which myocardial hyperemia is induced. This is achieved by continuous infusion of adenosine 140 µg/kg/min through a central venous catheter until steady-state maximum hyperemia is achieved after which pressure proximal (Pa) and distal to the stenosis (Pd) are measured simultaneously. FFR is calculated as the ratio of distal coronary pressure divided by proximal coronary pressure during steady-state maximum hyperemia. This procedure is the state-of-the-art operating procedure for performing FFR.

Patients with inducible myocardial ischemia were defined as having at least one coronary lesion with an FFR ≤0.75, while patients without inducible myocardial ischemia were defined as having no coronary lesion with an FFR ≤0.80. These cutoff values have been extensively validated [14]–[16]. To clearly demarcate ischemic status, patients with an intermediate FFR-value (0.76–0.80) were excluded from the analysis. Chronic total occlusions were arbitrarily assigned a FFR value of 0.50. In the remainder of the text patients with at least one FFR-value ≤0.75 will be referred to as FFR-positive, while patients with all FFR values >0.80 will be referred to as FFR-negative.

Coronary angiography was not performed in the healthy control group. Control subjects were randomly selected from local laboratory staff and had to meet the following criteria: age above 40, no previous vascular history, no medication use and absence of inflammatory or autoimmune disease. These subjects were questioned for coronary risk factors and symptoms of coronary artery disease. A routine electrocardiogram and standard blood analysis was performed as described below.

### Measurement of systemic markers of inflammation

After inclusion, blood was collected in lithium-heparin (LH) and ethylenediaminetetraacetic acid (EDTA) anticoagulated tubes. In the patient group blood samples were drawn immediately before angiography from the inserted arterial sheath while in healthy controls blood samples were obtained from a large antecubital vein.

Standard whole blood analysis, including complete blood cell count, renal function and lipid spectrum was performed in all participants.

### Cytokine measurements

Portions of 100 µl of whole blood LH – anticoagulated samples were transferred to 96 wells plates. 100 µl PBS was added and incubated at 37°C and atmosphere containing 5% CO2 for 2 hours. This was executed to allow valid comparison with the measurements of cell based parameters obtained with flow cytometry.

Afterwards, the samples were centrifuged for 5 minutes at 400×g, the supernatant was carefully transferred to sterile tubes and frozen at −80°C until further analysis. Cytokine levels of interleukine (IL)-6, IL-8 and tumor necrosis factor (TNF)-α were measured in these supernatants by Luminex cytometric bead analysis, according to the manufacturer's instructions. Undetectable values were imputed as 0.1 pg/ml. All levels of cytokines were corrected for total white blood cell count.

### Flow cytometry analysis

To assess expression of surface markers 50 µl of LH-anticoagulated whole blood was incubated with fluorescent antibodies against TRL2 (CD282 TLR2-FITC, Serotec, UK), TLR4 (CD284 TLR4-RPE, Serotec, UK), CD11b (CD11b PE-Cy7, Becton-Dickinson, NJ, USA), CD62L (CD62L ECD, Beckman Coulter, CA, USA) and CD14 (CD14 PC5, Beckman Coulter, CA,USA) for 30 minutes. After washing and whole blood lysis, the samples were subsequently analyzed by flow cytometry (Beckman Coulter FC 500). Granulocytes were gated based on their scatter properties. Monocytes were identified based on their scatter properties and positive CD14 staining. Expression of surface markers was quantified by mean fluorescense intensity (MFI).

### Severity of ischemic coronary disease and inflammation

To investigate a possible correlation between the severity of ischemic coronary disease and inflammatory markers we calculated Functional Syntax Score (FSS) for FFR-positive patients. FSS is a functional extension of the Syntax Score (a scoring system to grade anatomical severity of coronary artery disease) which has been shown to have predictive value in patients with multivessel disease and left main coronary artery disease treated with either PCI or CABG [17]. In this scoring system, each lesion is individually scored on the basis of location and morphological characteristics. The total score directly reflects the angiographic extent and severity of coronary artery disease. In a recent study, FSS showed superior predictive value over SS in FFR-guided PCI treated cohort [18]. Since all patients in our cohort were treated with an FFR-guided strategy, this scoring system is appropriate for this purpose. We hypothesized that increasing severity of the ischemic coronary disease would be associated with increased levels of inflammatory markers. To obtain FSS, Syntax score (SS) was calculated according to the instructions of the web-based Syntax Score Calculator (www.syntaxscore.com). Next, FSS was calculated by subtracting the individual scores of lesions with an FFR>0.80 from the total score. In this way, only hemodynamically significant lesions participate in the total score and thus reflect the severity of *ischemic* coronary artery disease (“ischemic burden”). The FFS scores of the FFR-positive group were divided into tertiles. We then compared inflammatory markers of the individual tertiles of FSS to inflammatory markers measured in the FFR-negative group and to each other.

### Statistical analysis

Results are presented as means±SD or medians (interquartile range; IQR) in case of a skewed distribution. Continuous variables were compared between groups with either student's T, Mann Whitney U-test in case of comparison between two groups and ANOVA or Kruskal-Wallis test or Jonckheere-Terpstra for comparison between multiple groups, as appropriate. Discrete variables were compared using Chi-square testing. A p value<0.05 was considered statistically significant. All analysis were performed using SPSS 17.0 (SPSS inc, Chicago, Ill, USA)

## Results

### Procedural results

In 108 patients with stable angina, FFR was measured and inflammatory markers were assessed. Twelve patients with an intermediate FFR-value (0.76–0.80) were not included in this analysis. Due to technical errors an additional 7 patients were excluded from further analysis. Consequently, 89 patients were analyzed in the current study. Demographics and history of patients according to ischemic status are presented in Table 1. Of the patients with an FFR≤0.75 included in this analysis 45 patients (76.3%) were treated with PCI, 9 (15.3%) with CABG and 5 (8.5%) treated conservatively. There were no statistically significant differences in risk profiles between the FFR-positive and FFR-negative patients, neither in age, sex, risk factors, body mass index or relevant cardiovascular history. The majority of patients had a normal Left ventricular ejection fraction (LVEF) (Table 1). In the healthy control group, 12 (75%) were male with a mean age of 49.8±5.6. None of the healthy controls used any medication. Five healthy control subjects (31.2%) reported a positive family history and 1 had known hyperlipidemia for which no medication was taken. None of the control subjects had any other relevant medical history. BMI of the healthy control subjects was lower (23.6±2.0 versus 27.1±4.3 and 27.6±3.4, p<0.01). Medication use in the patient group before undergoing catheterization and FFR-measurement is described in Table 2. Clopidogrel loading dose was administered depending on whether PCI was expected to be performed. Use of beta-blockers and administration of a loading dose clopidogrel was significantly higher in patients with lowest FFR≤0.75 (both p<0.01). Chronic use of clopidogrel, however, was similar in both groups (p = 0.83).

**Table 1:** Baseline Characteristics of patients with stable angina.

	FFR>0.80	FFR≤0.75	
	N = 30	N = 59	* p-value
Age	63.1±9.8	60.5±10.1	0.24
Sex - n male (%)	20 (66.7)	37 (62.7)	0.71
BMI	27.1±4.3	27.6±3.4	0.54
Risk factors- n (%)			
Smoking	5 (16.7)	17 (28.8)	0.21
Hypertension	13 (43.3)	27 (45.7)	0.83
DM	6 (20)	14 (23.7)	0.69
Hyperlipidemia	16 (53.3)	28 (47.5)	0.60
Family history	18 (60)	43 (72.9)	0.22
Peripheral artery disease	5 (16.7)	7 (11.8)	0.53
Cerebrovascular disease	1 (3.3)	1 (1.7)	0.62
Previous MI	6 (20)	11 (18.6)	0.88
Previous PCI	13 (43.3)	18 (30.5)	0.23
Previous CABG	1 (3.3)	1 (1.7)	0.62
Renal failure **	0 (0)	1 (1.6)	0.47
Normal LVEF (if known)	22 (73.3)	42 (71.2)	0.43
Mean lowest FFR	0.86±0.05	0.57±0.15	
White blood cell count (WBC)	6.49±1.51	6.84±1.73	0.34
Neutrophil count	4.05±1.06	4.46±2.69	0.43
Monocyte count	0.54±0.20	0.57±0.16	0.48

*Continuous values are presented as means ± standard deviation (SD). Categorical values are presented as number (percentages).*

* *Significance level 0.05. FFR = fractional flow reserve BMI = body mass index ;DM = diabetes mellitus; MI = myocardial infarction; PCI = percutaneous coronary intervention; CABG = coronary artery bypass grafting; LVEF = left ventricular ejection fraction.*

** *Defined as a serum creatinin >150 µmol/l.*

**Table 2:** Medication use of patients with stable angina at inclusion.

	FFR>0.80	FFR≤0.75	
Medication – n(%)	N = 30	N = 59	p-value*
ASA	24 (80)	55 (93)	0.08
β-blocker	18 (60)	51 (86)	**<0.01**
ACE-inhibitor	8 (26.7)	15 (25.4)	0.90
Statin	28 (93.3)	54 (91.5)	0.77
Clopidogrel (chronic use)	9 (30)	19 (32.2)	0.83
Clopidogrel (loading dose)	3 (10)	32 (54.2)	**<0.01**

*Proportions were compared using Chi-square testing.*

* *Significance level 0.05. FFR = fractional flow reserve; ASA = Acetylsalicylic acid; ACE = angiotensin-converting enzyme.*

### White blood cell (WBC) count

In the group of patients with stable angina total WBC count, neutrophil and monocyte-counts did not differ between FFR-positive and FFR-negative patients, as shown in Table 1. Patients with stable angina had significantly higher total WBC, neutrophil and monocyte counts than healthy controls, respectively 6.72±1.66×10^9^/l versus 5.37±1.35×10^9^/l leukocytes, p<0.01, 4.32±2.23×10^9^/l versus 2.99±1.11×10^9^/l neutrophils, p<0.01 and 0.56±0.18×10^9^/l versus 0.43±0.12×10^9^/l monocytes, p<0.01.

### Cytokine concentrations

**Figure 1:**
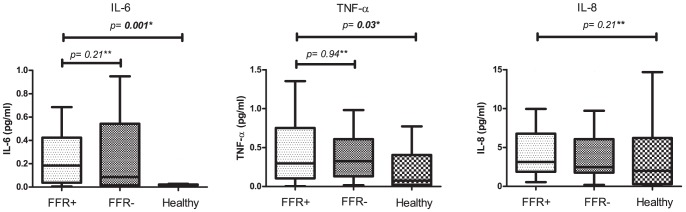
Comparison of levels of cytokines for FFR-positive and FFR-negative patients and healthy controls. *Comparison by Kruskal-Wallis test.** Between subgroup differences compared with Mann Whitney U test.

Concentrations of IL-6, IL-8 and TNF-α were measured in the supernatants of all patients and healthy controls and corrected for total white blood cell count. In 2 patients cytokine-concentration could not be measured, due to technical reasons. Concentrations of the above mentioned cytokines were compared among groups. Results are depicted in Figure 1. We did not observe differences in concentrations for any of the cytokines in supernatants of FFR-positive patients compared to those of FFR-negative patients. Median concentration (IQR) of IL-6 in FFR-positive patients compared to FFR-negative patients was 0.19 (0.04–0.45) versus 0.09 (0.02–0.54), p = 0.21, concentration of IL-8 was 3.4 (1.9–7.2) versus 2.7 (1.8–6.9), p = 0.56 and concentration of TNF-α 0.30 (0.10–0.75) versus 0.34 (0.14–0.66), p = 0.94. We did observe a significantly lower concentration of both IL-6 and TNF-α in the healthy controls compared to the patients with stable angina (p = 0.001 and p = 0.03, respectively).

Concentration of IL-8 in the supernatants did not significantly differ between all three groups (p = 0.21).

### Expressions of TLR2, TLR4, CD14, CD11b and CD62L on peripheral blood cells

**Figure 2:**
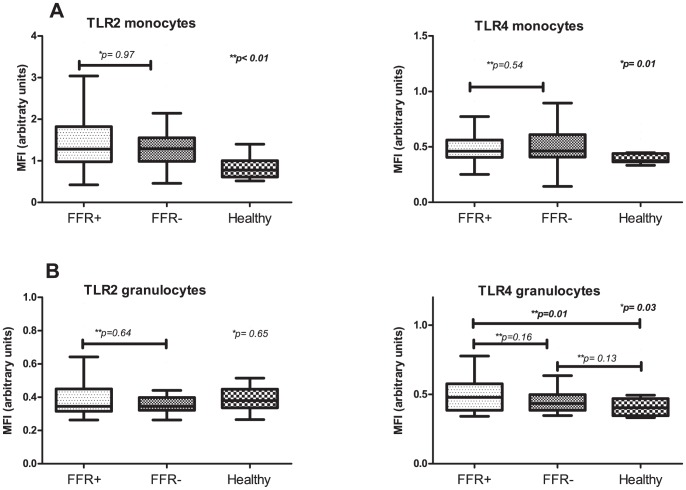
Expression levels of TLR2 and TLR4 on monocytes (A) and granulocytes (B) FFR-positive and FFR-negative patients and healthy control subjects. *Comparison by Kruskal-Wallis test for all groups.** Between subgroups with Mann Whitney U test. TLR = toll-like receptor.

Expression of TLR2 and TLR4 quantified by mean fluorescence intensity (MFI), was measured by flow cytometry on monocytes and granulocytes in blood samples of all 89 patients. We did not observe any significant differences between the expression levels for TLR2 and TLR4 on both monocytes (Figure 2A) and granulocytes (Figure 2B) between FFR-positive and FFR-negative patients. TLR2 and TLR4 expression on monocytes and TLR4 expression on granulocytes in healthy control subjects was significantly lower compared to both FFR-positive and FFR-negative patients (p<0.01, p = 0.01 and p = 0.03).

**Figure 3:**
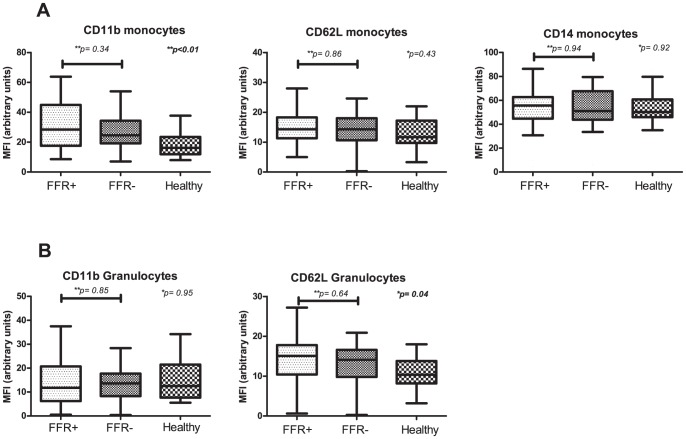
Expression levels of CD11b, CD62L and CD14 on monocytes (A) and CD11b and CD62L on granulocytes (B) in FFR-negative patients, FFR-positive and healthy control subjects. *Comparison by Kruskal-Wallis test for all groups.** Between subgroups with Mann Whitney U test.

We then compared expression levels of CD11b and CD62L on both monocytes and granulocytes and CD14 on monocytes (Figure 3A and 3B). We observed no differences between the FFR-groups in expression of CD11b on monocytes (p = 0.34) or granulocytes (p = 0.85). Expression of CD62L on both monocytes and granulocytes also did not differ between FFR-positive and FFR-negative patients (p = 0.86 and p = 0.64, respectively). Healthy control subjects had significantly lower expression levels of CD11b on monocytes (p<0.01) and CD62L on granulocytes (p = 0.04). CD11b on granulocytes and CD62L on monocytes did not differ between groups (p = 0.95 and p = 0.43 respectively). Moreover, no difference in expression of CD14 on monocytes could be detected (p = 0.92).

### Influence of severity of ischemic coronary disease on inflammatory markers

**Figure 4:**
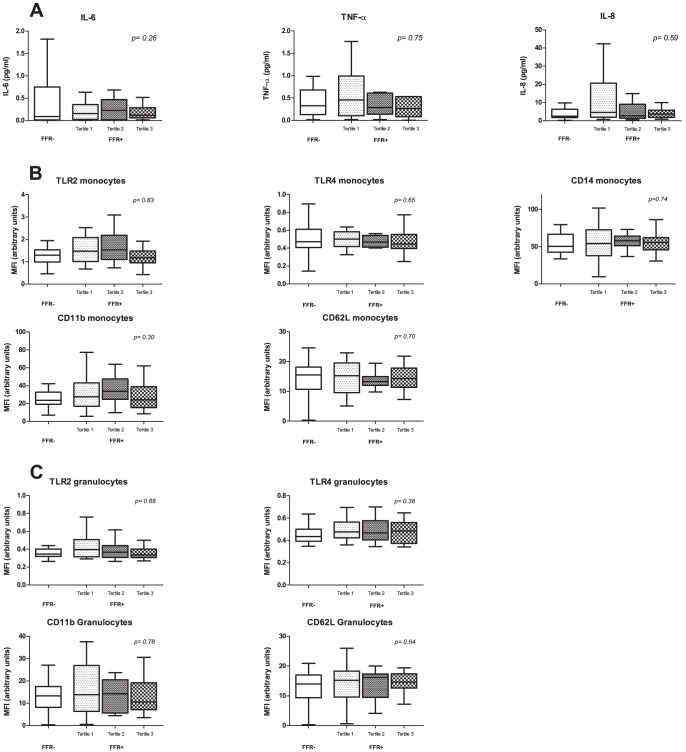
Concentrations of cytokines and surface markers on monocytes and granulocytes according to FSS tertiles and FFR-negative patients. Concentrations of cytokines (A) and surface markers on monocytes (B) and granulocytes (C) according to FSS tertiles (of FFR-positive patients) and FFR-negative patients. Comparison of all subgroups was performed with the Jonckheere-Terpstra test. FSS = functional syntax score.

To assess the influence of the *severity and extent of ischemic coronary disease* on the measured markers of inflammation we calculated FSS for each FFR-positive patient (as described above), divided FSS-scores into tertiles (range 1 to 28, intertertile range 7 to 14) and compared inflammatory markers of each tertile to each other and to FFR-negative patients. No between-subgroup differences were noted (p-values all >0.05). More importantly, there was no incremental relation between FSS tertiles and the measured markers (Figure 4A–C).

## Discussion

The presence of inducible myocardial ischemia is of key importance for the prognosis of stable coronary artery disease. Numerous studies have shown increased rates of myocardial infarction and cardiovascular death when substantial myocardial ischemia is present as measured by FFR. Systemically measured inflammatory markers have also been associated with adverse outcome in patients suffering from cardiovascular disease. The origin of these inflammatory parameters can be the local inflammatory unstable plaque, can be a reflection of the atherosclerotic burden in the vascular system or an epi-phenomenon. Since plasma levels of pro-inflammatory cytokines as well as cell-based markers on circulating cells have been found to be elevated in acute coronary syndromes, and the presence and extent of inducible ischemia associated with increased occurrence of myocardial infarction and death, we hypothesized that inducible ischemia is associated with increased levels of blood derived inflammatory markers.

In this study, however, we found no relation between the investigated inflammatory markers and the presence of inducible myocardial ischemia as evidenced by FFR. In line with previous reports, we observed a difference in inflammatory markers between healthy subjects and patients with stable angina, pointing to a chronically elevated inflammatory status in patients with coronary artery disease [19]. Our observations suggest that this elevated inflammatory state is not altered by the presence of inducible ischemia by local atherosclerotic luminal narrowing, but probably a reflection of co-morbidities or systemic risk factors with concomitant generalized atherosclerotic disease, independently of its functional severity.

The latter is supported by the baseline characteristics that reveal that the FFR-positive and negative patient groups have comparable atherosclerotic risk profiles and history of previous MI or PCI as opposed to the healthy control group, which has virtually no risk factors and no history of coronary pathology. This study therefore shows that local lesions that may induce local ischemia do not explain the increased inflammatory blood profile in patients suffering from stable coronary artery disease. Even if the extent and severity of ischemic coronary disease is taken into account according to FSS, no relationship between the inflammatory markers and inducible ischemia could be identified.

Fractional Flow Reserve (FFR) is the most reliable method for assessing the ischemic potential of coronary stenoses. Reducing myocardial ischemia is associated with improved prognosis [20] and by use of FFR it is possible to adequately pinpoint which lesions are responsible for ischemia. FFR-guided revascularization provides optimal relief of ischemia, preventing unnecessary stenting or bypass surgery of non-ischemia causing lesions while at the same time preventing undertreatment of lesions that appear moderate on angiography but do cause ischemia [21].

In this study, we investigated a specific subset of inflammation markers known to be associated with coronary artery disease. Toll-like receptors (TLRs) are a key part of the innate immune system and have been shown to play an important role in both the initiation and progression of coronary disease [22], [23]. Increased expression of TLR4 on circulating monocytes and increased transcription of pro-inflammatory cytokines and co-stimulatory molecules, reflecting downstream activation of TLR stimulation, have been found in patients with unstable angina (UA) and acute myocardial infarction (AMI) compared to patients with stable angina and healthy controls [24], [25].

CD11b, also known as Integrin αM, is involved in adhesion and transendothelial migration an early marker for cellular activation of leukocytes [26]. Furthermore, CD11b-expression has been shown to be upregulated immediately after stimulation of TLRs [27]. CD62L (L-selectin) is a member of the selectin family, expressed on both monocytes and granulocytes and is involved in cell adhesion [28]. Both granulocytes and monocytes readily shed CD62L upon stimulation.

It has been shown in both experimental and clinical settings that short lasting myocardial ischemia elicits an inflammatory response. In a porcine experiment 30 minutes of induced ischemia resulted in marked increase of plasma levels of IL-6 and TNF-α [29]. In patients with coronary artery disease, dobutamine-induced ischemia resulted in an increase in plasma levels of IL-6 and tissue factor. Furthermore, IL-6 levels were related to left ventricular dysfunction at peak stress and rate of recovery of left ventricular function [30]. In patients with a reversible perfusion defect in myocardial perfusion imaging after stress, TLR mediated leukocyte activation was attenuated [31]. These findings provide both experimental as well as clinical evidence that an acute inflammatory response to ischemic episodes occurs which is detectable in peripheral blood samples. However, these results pertain to acutely induced ischemia and it is unknown whether inflammatory status is permanently altered by short repetitive ischemic episodes. In this study we found no evidence for this.

## Limitations

Due to the fact that FFR measurements are obviously unknown prior to angiography, patient groups are inevitably unequal in size. Also, FFR-positive patients were more frequently pre-treated with a loading dose of clopidogrel. Clopidogrel has been shown to have anti-inflammatory properties [32], and thus it is possible that inflammatory status in the FFR-positive group could be underestimated by pre-treatment. It must be noted however, that any anti-inflammatory properties of clopidogrel have mainly been observed during chronic treatment [32]–[35], and chronic clopidogrel treatment did not differ between the FFR positive and negative groups. Quinn et al specifically investigated effects of clopidogrel treatment (>24 hours) before PCI on inflammatory markers and observed that serum CD40 ligand and IL-6 were not affected by clopidogrel pre-treatment [36]. It is therefore unlikely that pre-treatment with a loading dose of clopidogrel would have played a significant role in our study. In any case, we feel withholding clopidogrel pretreatment in patients if stenting is anticipated is unethical and potentially hazardous. Over 90% of the patients with stable angina in this study are treated with statins (HMG-CoA reductase inhibitors), which have been shown to have anti-inflammatory properties [37]–[39]. Despite the use of statins, inflammatory markers are increased in the patient groups as compared to the healthy controls. In the patient groups it is conceivable that use of statins may mask small differences related to the presence or absence of inducible ischemia. Blood samples were taken from an arterial sheath in the patient groups. Consequently, no statements can be made on possible differences in other locations of the vascular system (e.g. coronary artery or coronary sinus) in these patients. In the healthy control group, blood was obtained from an antecubital vein. Although differences in inflammatory markers between locally (coronary sinus) obtained blood and peripheral blood in coronary artery disease have been observed [40], [41], to our knowledge differences of these markers between *peripheral* venous and *peripheral* arterial blood have not been reported [42]. BMI of the healthy control subjects was significantly lower than that of the patient groups. As increased levels of inflammatory markers have been reported in obese subjects [43]–[45], we cannot exclude the possibility that this may have influenced biomarker levels in the patient groups, although BMI was only moderately increased in the FFR positive and FFR-negative patients. In this study we only analyzed a specific subset of inflammatory markers; IL-6, IL-8 and TNF-α as inflammatory cytokines and CD11b, CD62L, TLR2 and TLR4 as markers for cellular activation of circulating cells. Although we investigated cell based expression as well as secreted markers for inflammation, we cannot discount the possibility that repetitive ischemia exerts effects through different mechanisms and is reflected by different biomarkers than the ones investigated here.

### Conclusions

Inducible myocardial ischemia is not associated with increased concentrations of IL-6, IL-8 and TNF-α in blood or expression of CD11b, CD62L, CD14, TLR2 or TLR4 on circulating leukocytes in patients with stable angina.
